# Necroptosis and ferroptosis are alternative cell death pathways that operate in acute kidney failure

**DOI:** 10.1007/s00018-017-2547-4

**Published:** 2017-05-27

**Authors:** Tammo Müller, Christin Dewitz, Jessica Schmitz, Anna Sophia Schröder, Jan Hinrich Bräsen, Brent R. Stockwell, James M. Murphy, Ulrich Kunzendorf, Stefan Krautwald

**Affiliations:** 1 Department of Nephrology and Hypertension, University Hospital Schleswig–Holstein, Campus Kiel, Georges-Köhler-Haus, Fleckenstr. 4, 24105 Kiel, Germany; 2 Department of Pathology, University of Hannover, 30625 Hannover, Germany; 3 Department of Biological Sciences and Department of Chemistry, Columbia University of New York, New York, NY 10027 USA; 4 The Walter and Eliza Hall Institute of Medical Research, Parkville, VIC 3052 Australia; 5 Department of Medical Biology, University of Melbourne, Parkville, VIC 3052 Australia

**Keywords:** Ferroptosis, ACSL4, Necroptosis, MLKL, Ischemia-reperfusion injury

## Abstract

Ferroptosis is a recently recognized caspase-independent form of regulated cell death that is characterized by the accumulation of lethal lipid ROS produced through iron-dependent lipid peroxidation. Considering that regulation of fatty acid metabolism is responsible for the membrane-resident pool of oxidizable fatty acids that undergo lipid peroxidation in ferroptotic processes, we examined the contribution of the key fatty acid metabolism enzyme, acyl-CoA synthetase long-chain family member 4 (ACSL4), in regulating ferroptosis. By using CRISPR/Cas9 technology, we found that knockout of *Acsl4* in ferroptosis-sensitive murine and human cells conferred protection from erastin- and RSL3-induced cell death. In the same cell types, deletion of mixed lineage kinase domain-like (*Mlkl*) blocked susceptibility to necroptosis, as expected. Surprisingly, these studies also revealed ferroptosis and necroptosis are alternative, in that resistance to one pathway sensitized cells to death via the other pathway. These data suggest a mechanism by which one regulated necrosis pathway compensates for another when either ferroptosis or necroptosis is compromised. We verified the synergistic contributions of ferroptosis and necroptosis to tissue damage during acute organ failure in vivo. Interestingly, in the course of pathophysiological acute ischemic kidney injury, ACSL4 was initially upregulated and its expression level correlated with the severity of tissue damage. Together, our findings reveal ACSL4 to be a reliable biomarker of the emerging cell death modality of ferroptosis, which may also serve as a novel therapeutic target in preventing pathological cell death processes.

**Electronic supplementary material:**

The online version of this article (doi:10.1007/s00018-017-2547-4) contains supplementary material, which is available to authorized users.

## Introduction

Apoptosis, a form of cell death triggered by proteases of the caspase family, had long been considered synonymous with regulated cell death (RCD), whereas necrosis was thought to occur predominantly in an accidental manner. However, this paradigm has been challenged by numerous recent studies, which demonstrated that necrotic signaling pathways can also occur in a highly regulated and genetically controlled manner [1]. To date, necroptosis—originally defined as being dependent on the receptor interacting protein kinase 1 (RIPK1)—is the most thoroughly examined form of regulated necrosis, executed by RIPK3 and its substrate, the pseudokinase mixed lineage kinase domain-like protein (MLKL). The precise mechanism by which MLKL induces membrane rupture and ultimately causes cell death remains to be definitively established [2–4]. However, RIPK3-mediated phosphorylation of the activation loop in MLKL has been suggested to trigger a molecular switch to induce necroptotic cell death [5]. Consistent with an immune-activating role for this form of cell death, necroptosis is characterized by cellular swelling, rapid membrane permeabilization and concomitant release of damage-associated molecular patterns (DAMPs) into the extracellular space. Therefore, necroptosis has been implicated in the development of a range of autoimmune, neurodegenerative and inflammatory diseases, such as acute pancreatitis and ischemic injury, among others [6].

In contrast, ferroptosis is a recently recognized iron- and reactive oxygen species (ROS)-dependent form of RCD that is remarkably distinct from necroptosis and other forms of RCD at genetic, biochemical and morphological levels [7]. Ferroptotic cell death is characterized by lipid peroxidation: a process is negatively regulated by the cystine-glutamate antiporter system *X*_c_^−^, which provides the cysteine used for glutathione synthesis. Glutathione is crucial for the activity of glutathione peroxidase 4 (GPX4), an enzyme that protects cells against lipid oxidation. Accordingly, in vivo studies in mice confirmed that the activity of GPX4 is essential to prevent ferroptosis [8]. An additional requirement for cells to undergo ferroptosis is the presence of polyunsaturated fatty acids (PUFAs), including arachidonic acid (AA, 20:4), which are susceptible to peroxidation, leading to the formation of lipid hydroperoxides [9]. Recently, it was shown that dysregulation of lipid metabolism is associated with ferroptosis, but it has remained unclear which genes precisely confer resistance to ferroptosis. Using insertional mutagenesis of haploid KBM7 cells, a recent study revealed that the deletion of two genes, lysophosphatidylcholine acyltransferase 3 (*Lpcat3*) and acyl-CoA synthetase long-chain family member 4 (*Acsl4*), suppress ferroptosis by limiting the membrane-resident pool of oxidation-sensitive fatty acids [10]. Mechanistically, it is expected that the execution of ferroptosis can only proceed when highly oxidizable PUFAs such as AA are present at sufficient concentration in target cell membranes. Indeed, during the finalization of this manuscript, an important role for Acls4 in modifying the plasma membrane lipidome in ferroptosis was reported [11]. However, mechanistic details of the events in ferroptotic cell death that occur downstream of PUFA peroxidation are yet to be elucidated.

RCD is either immunologically silent or immunogenic. Immunogenic cell death by regulated necrosis causes extensive tissue damage in a wide variety of diseases, including sepsis, stroke, myocardial infarction, ischemia-reperfusion injury (IRI) and solid organ transplantation. We have found previously that necroptosis and ferroptosis represent two different modes of regulated necrosis that mediate IRI in mice [12]. Nevertheless, it remains of interest whether inter-pathway cross-talk contributes to regulation of these two cell death pathways, their associated pathologies in common clinical models, and whether dual targeting of both pathways by combination therapies may be necessary for effective clinical intervention.

In the present study we found that the deletion of *Acsl4*, an essential gene of lipid metabolism, confers resistance to ferroptosis in both murine and human cells, suggesting that ACSL4 expression and mutation might serve as a biomarker to aid in predicting sensitivity to ferroptosis. Interestingly, genetic and pharmacological suppression of ferroptosis in these cells leads to a time- and concentration-dependent hypersensitization to necroptosis. Reciprocally, we confirmed the coordinated regulation of these two different pathways of regulated necrosis by deletion of *Mlkl*, an essential mediator of RIPK3-initiated necroptosis, which led to necroptosis-insensitive cells that were more susceptible to ferroptosis. Again, this scenario was time- and concentration-dependent and was recapitulated by pharmacological inhibition of necroptosis in the genetically unmodified parental cell line. Subsequently, we verified these in vitro data in a functionally relevant in vivo model of IRI. *Mlkl*-knockout animals exhibited significantly increased ACSL4 protein expression within the first 24 h after reperfusion compared to the wildtype counterparts, indicating that defective necroptotic signaling in this model switches the etiopathology at the onset towards enhanced ferroptosis. Further, we observed an increased expression of ACSL4 in human kidney biopsies from patients with acute tubular injury (ATI) following kidney transplantation and severe thrombotic microangiopathy of native kidney, suggesting that ACSL4 abundance might also serve as a pharmacodynamic marker of ferroptosis execution. Overall, these data support the ideas that the interplay between the ferroptosis and necroptosis cell death pathways is crucial to pathophysiological acute kidney failure and that both pathways contribute to the total organ damage. In parallel and independent of our study, other groups have drawn similar conclusions, consistent with our findings, that ACSL4 is a potential biomarker and contributor to ferroptosis [11, 13].

Here, we have found that ACSL4 is both a predictive biomarker and pharmacodynamic marker of the regulated cell death modality of ferroptosis in vivo in acute kidney failure, thereby providing an important platform for clinical monitoring and diagnosis of ferroptosis-mediated pathologies.

## Materials and methods

### Cell culture

NIH3T3, HT-29, HT-1080 and L929 cells were originally obtained from ATCC and were cultured in Dulbecco’s Modified Eagle’s Medium (DMEM) (Gibco/Thermo Fisher Scientific, Darmstadt, Germany) supplemented with 10% (vol/vol) FCS, 100 U/mL penicillin, and 100 μg/mL streptomycin. HT-1080 medium was additionally supplemented with non-essential amino acids (100× solution purchased from Thermo Fisher Scientific). All cell lines were cultured in a humidified 5% CO_2_ atmosphere.

### Reagents and antibodies

Recombinant purified TNFα, the annexin V-FITC antibody and the 7-AAD antibody were purchased from BioLegend (London, United Kingdom). Necrostatin-1_s_ (Nec-1_s_) was obtained from BioVision, Inc. (Milpitas, CA, USA). The zVAD-fmk (herein referred to as zVAD) was purchased from Bachem (Weil, Germany). Erastin (era), GSK’872, Necrosulfonamide (NSA) and the anti-MLKL antibody (clone 3H1) were obtained from Calbiochem (Merck Millipore, Darmstadt, Germany). Synthesis of the ferrostatin derivative 16-86 and the ferroptosis inducer RSL3 were described previously [12, 14]. Ferrostatin-1 (Fer-1) was purchased from Xcess Biosciences Inc. (San Diego, CA, USA). Dabrafenib was purchased from Selleckchem (Absource Diagnostics, Munich, Germany). GW806742X was purchased from Biomol (Hamburg, Germany). The monoclonal anti-ACSL4, anti-GPX4, the anti-human and anti-mouse monoclonal phospho-MLKL antibodies were all obtained from Abcam (Cambridge, United Kingdom). The β-actin antibody was purchased from Cell Signaling (Frankfurt, Germany).

### Generating stable CRISPR/Cas9 knockout cell lines

**Fig. 1:**
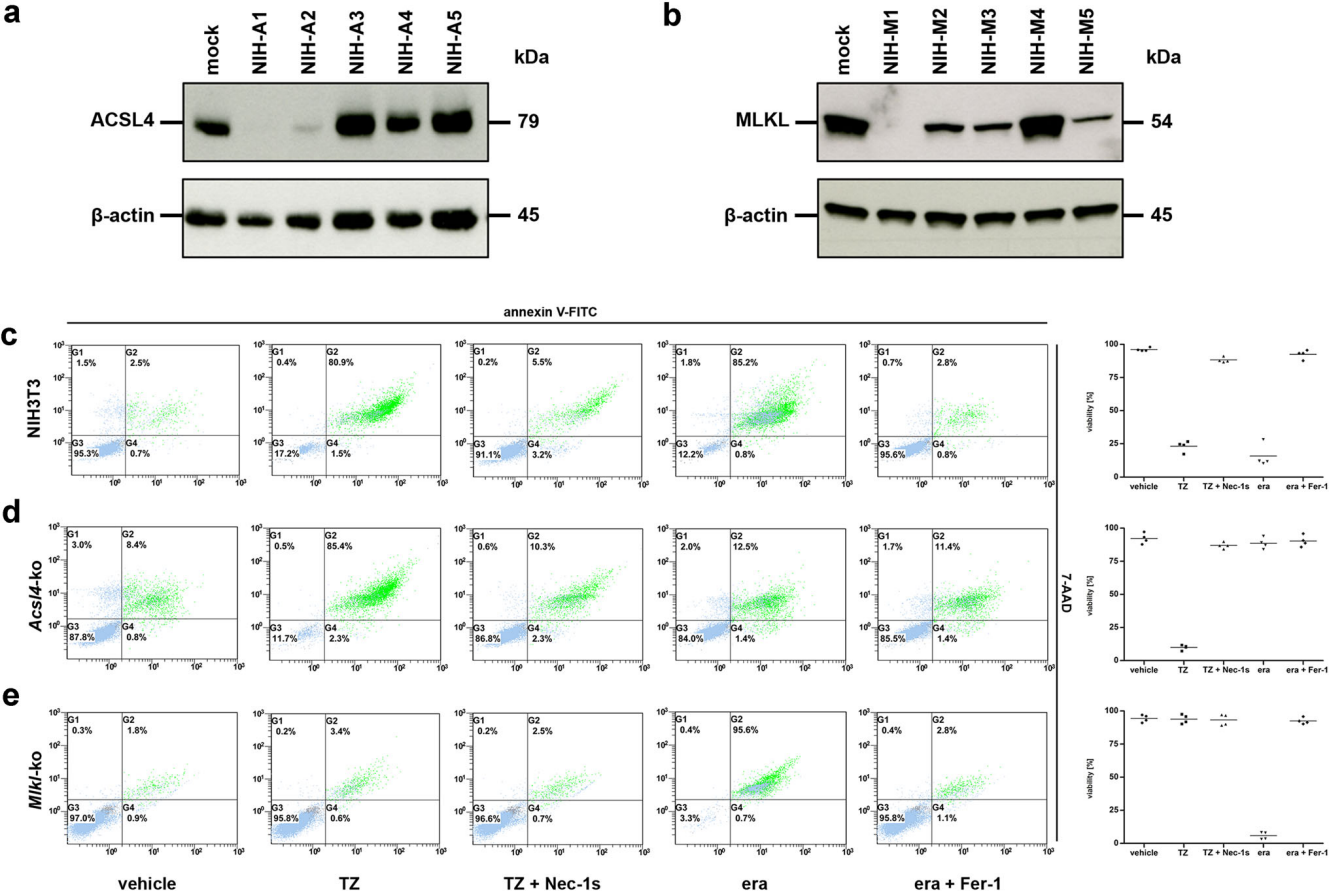
*Acsl4*- and *Mlkl*-knockout NIH3T3 cells are protected from ferroptosis and necroptosis, respectively. **a** Five different *Acsl4*-silenced clones, referred to as NIH-A1 to NIH-A5, and **b** five different *Mlkl*-silenced clones, referred to as NIH-M1 to NIH-M5, were analyzed by Western blotting for the ablation of the indicated target genes. Lysates of mock-transfected (non-edited) control cells were plotted in each case on the left lane. Both blots were redeveloped with an antibody against β-actin as a loading control. For all assays shown subsequently we used the NIH3T3 clones NIH-A1 (for *Acsl4*-ko) and NIH-M1 (for *Mlkl*-ko), respectively. FACS analysis for the necrotic marker 7-AAD and phosphatidylserine exposure (annexin V-FITC positivity) in **c** mock-transfected (non-edited), **d** *Acsl4*-edited, and **e** *Mlkl*-edited NIH3T3 cells which were treated for 16 h at 37 °C with DMSO (vehicle), 100 ng/ml TNFα + 25 µM zVAD (TZ), 100 ng/ml TNFα + 25 µM zVAD + 50 µM necrostatin-1_s_ (TZ + Nec-1_s_), 10 µM erastin (era), and 10 µM erastin + 1 µM ferrostatin-1 (era + Fer-1) as indicated. Necroptosis was blocked by addition of Nec-1 _s_, and ferroptosis blocked by Fer-1. **c**–**e** FACS dot plots of one representative experiment are shown, with adjacent *box plots* presenting the mean and standard deviation of four independent experiments

All sgRNAs for the selected targets (*Acsl4* and *Mlkl*) were designed in silico via the CRISPR Design Tool (http://tools.genome-engineering.org). The single-stranded sgRNA oligos were annealed and ligated into an expression plasmid bearing both sgRNA scaffold backbone and Cas9 (pX330-U6-Chimeric_BB-CBh-hSpCas9, Addgene Plasmid No. 42230). The resulting plasmid (annotated as pSpCas9_sgRNA) was then co-transfected with the pcDNA3.1(+) vector (Invitrogen, No. V790-20) containing a geneticin resistance gene into target cells. After single-cell cloning by serial dilution in 96-well plates (media was supplemented with 1 mg/ml G418) the clones were assayed and selected for their target gene-knockout by Western blot analysis to identify stable CRISPR/Cas9-ko cell lines as illustrated in Fig. 1a, b and Supplementary Material, Fig. S1a, S1b. To exclude feasible off-target effects or clonal variations within the cell population we generated and analyzed three different guide RNAs for each target gene and species (*Acsl4* and *Mlkl*, respectively and observed congruent outcomes in each case. Nevertheless, to avoiding confusion we have presented data obtained using single cell clones, if not indicated otherwise. The chosen sequences for these different synthesized sgRNAs are listed subsequently (m = targeting gene of interest in murine NIH3T3 and L929 cells, h = targeting gene of interest in human HT-1080 and HT-29 cells):

m*Mlkl*_1.1: GCACACGGTTTCCTAGACGCTGG

m*Mlkl*_1.2: GACTTCATCAAAACGGCCCAGGG

m*Mlk*l_3.1: AGGAACATCTTGGACCTCCGTGG

h*Mlkl*_1.1: AAGAAACAGTGCCGGCGCCTGGG

h*Mlkl*_1.2: CACACCGTTTGTGGATGACCTGG

h*Mlkl*_1.3: GGAGCTCTCGCTGTTACTTCAGG

m*Acsl4*_1.1: ACAGAGCGATATGGACTTCCAGG

m*Acsl4*_1.2: CTAGCTGTCATAGACATCCCTGG

m*Acsl4*_3.1: GATTACTAGTGTTGAGCTTCTGG

h*Acsl4*_1.1: CTAGCTGTAATAGACATCCCTGG

h*Acsl4*_2.1: TGCAATCATCCATTCGGCCCTGG

h*Acsl4*_3.1: GATTACCAGTGTTGAACTTCTGG

### Detection of knockout clones and analysis of cell death by Western blotting

For immunoblotting, cells were lysed in ice-cold 10 mM Tris–HCl, pH7.5, 50 mM NaCl, 1% Triton X-100, 30 mM sodium pyrophosphate, 50 mM NaF, 100 μM Na_3_VO_4_, 2 μM ZnCl_2_, and 1 mM phenylmethylsulfonyl fluoride (modified Frackelton buffer). Insoluble material was removed by centrifugation (14,000×*g*, 10 min, 4 °C), and protein concentration was determined using a commercial Bradford assay kit according to the manufacturer’s instructions (Bio-Rad, Munich, Germany). Equal amounts of protein (20 μg per lane) were resolved by reducing SDS/PAGE and transferred to a nitrocellulose membrane (GE Healthcare Life Sciences, Freiburg, Germany). Western blots were performed using specified primary antibodies and corresponding secondary horseradish peroxidase-linked polyclonal anti-rabbit antibody from abcam (Berlin, Germany). Immune complexes were visualized by enhanced chemiluminescence (ECL) from GE Healthcare Life Sciences.

### Assessment of cell death in vitro

Phosphatidylserine exposure to the outer cell membrane of apoptotic cells or at the inner plasma membrane of necrotic cells and incorporation of 7-AAD into necrotic cells was quantified by fluorescence-activated cell sorting (FACS) analysis. Stainings were performed according to the manufacturer’s instructions (BioLegend). Fluorescence was analyzed using an FC-500 (Beckman Coulter, Krefeld, Germany) flow cytometer.

To determine the release of lactate dehydrogenase (LDH) from damaged cells the CytoTox 96 Non-Radioactive Cytotoxicity Assay was used according to the manufacturer’s instructions (Promega, Mannheim, Germany). The LDH activity was measured by colorimetry at an absorbance of 490 nm on a Sunrise™ plate reader (Tecan, Crailsheim, Germany) and the percent LDH release [LDH release (%) = 100 × experimental LDH release (OD490)/maximum LDH release (OD490)] was calculated from the average of three identically treated wells.

### Analysis of reactive oxygen species (ROS) production

Parental and *Mlkl*-edited NIH3T3 cells were treated with 10 µM Erastin for the indicated times and harvested by trypsinization. Thereafter, the cells were suspended in 500 µl PBS containing 2 µM BODIPY^®^ (581/591) C11 (Gibco/Thermo Fisher Scientific, Darmstadt, Germany). Afterwards, cells were incubated for 10 min at 37 °C in a tissue culture incubator, resuspended in 700 µl of fresh PBS, strained through a 40 µM cell strainer (BD Biosciences, Heidelberg, Germany), and analyzed using a flow cytometer (FC-500 from Beckman Coulter, Krefeld, Germany) equipped with 488 nm laser for excitation. Data were collected from the FL1. A minimum of 10,000 cells were analyzed per condition.

### Mice

All male mice reported in this study were on C57BL/6 background and carefully matched for age, sex and weight. For our analyses animals were used in an age of 8 weeks. The *Mlkl*-knockout mice have been described previously [15] and were bred and co-housed in Kiel. All mice were kept on a standard diet and a 12 h day night rhythm. All in vivo experiments were performed according to the Protection of Animals Act after approval of the German local authorities.

### Ischemia-reperfusion injury (IRI)

Induction of murine kidney IRI was performed via a midline abdominal incision and a bilateral renal pedicle clamping for 35 min using microaneurysm clamps (Aesculab, Inc. Center Valley, PA, USA). Throughout the surgical procedure, the body temperature was maintained between 36 and 37 °C by continuous monitoring using a temperature-controlled self-regulated heating system (Fine Science Tools). After removal of the clamps, reperfusion of the kidneys was confirmed visually. The abdomen was closed in two layers using standard 6-0 sutures. To maintain fluid balance, all of the mice were supplemented with 1 ml of prewarmed phosphate buffered saline (PBS) administered intraperitoneally directly after surgery. Mice were sacrificed after different reperfusion time points as indicated in the text. All ischemia-reperfusion experiments were performed in a double-blinded manner. Where indicated, 16–86 (*c* = 2.0 mg/kg body weight) was applied intraperitoneally 15 min before the onset of ischemia and additionally every 3 h for 24 h in a final volume of 300 μL, respectively. In those experiments, control mice received 300 μL of vehicle (1.5% DMSO in PBS) at each time point.

### Histology, immunohistochemistry, and morphological assessment

Kidney biopsies were fixed in 4% neutral buffered formaldehyde and embedded in paraffin. 3 μm sections were dewaxed, rehydrated and stained by periodic acid-Schiff (PAS) according to routine protocols. Tubular damage was graded (no, mild, moderate, severe damage) in blinded samples by an experienced renal pathologist. Antigen retrieval for phospho-MLKL and ACSL4 was performed in 0.01 M sodium citrate buffer (pH 6.0), for MLKL Tris–EDTA buffer (pH 9.0) (Zytomed Systems, Berlin, Germany) for 30 min at 98 °C was used followed by 10 min 3% H_2_O_2_ to block endogenous peroxidase activity. Mouse sections were incubated 1:50 with mouse-specific monoclonal rabbit phospho-MLKL (phospho S345) antibody (clone EPR9515; abcam), monoclonal rat MLKL (clone 3H1; Merck) 1:100 and monoclonal rabbit anti-ACSL4 antibody (clone EPR8640; abcam) 1:500 for 60 min. For MLKL stains HRP-conjugated polyclonal donkey anti-rat immunoglobulin (dianova, Hamburg, Germany) and for both ACSL4 and phospho-MLKL stains HRP-conjugated polyclonal goat anti-rabbit immunoglobulin (Jackson ImmunoResearch Laboratories, Inc., West Baltimore Pike, PA, USA) were used 1:100 as secondary antibodies for 30 min followed by 3,3′-diaminobenzidine detection. In human tissues human-specific monoclonal rabbit to phospho-MLKL (phospho S358) (clone EPR9514; abcam) was used 1:50 for 60 min. The detection system for phospho-MLKL and ACSL4 in human samples consisted of ZytoChem Plus HRP Polymer System (Mouse/Rabbit) and 3,3′-diaminobenzidine (Zytomed Systems) according to the manufacturer´s recommendations. For negative controls, primary antibodies were omitted. Subsequently sections were mildly counterstained with hemalum. Sections were evaluated using an Olympus U-DO3 microscope. Representative photomicrographs were taken using a Zeiss system (Axioplan microscope with MRT digital camera and Axiovision Software; Zeiss, Oberkochen, Germany).

### Statistical methods and analyses

For all experiments, differences of datasets were considered statistically significant when *p* values were lower than 0.05, if not otherwise specified. Statistical comparisons were performed using the two-tailed Student’s *t* test. Asterisks are used in the figures to specify statistical significance (*p < 0.05; **p < 0.02; ***p < 0.001). Statistics are indicated as SD unless otherwise specified.

### Sample collection of human kidney biopsies

Ethical approval has been obtained from the local authorities, AZ: D415/14. All patients gave informed written consent.

## Results

### Constitutive deletion of *Acsl4* and *Mlkl* protects these cells from ferroptosis and necroptosis, respectively, in different murine and human cell lines

In investigations of the role of regulated cell death scenarios in renal diseases, we have found previously that cyclophilin D-mediated mitochondrial permeability transition (MPT) and receptor-interacting protein kinase (RIPK)-1 and RIPK3-mediated necroptosis mediate common ischemia-reperfusion injury in vivo [16]. The role of ferroptosis in this context was subsequently illustrated by different studies (reviewed in [17] and our own data suggesting that this mode of cell death is additionally involved in IR-mediated organ damage [12]. It remains of interest to establish the extent to which these two pathways intersect, especially because an interconnection between these different pathways may represent a common target for therapeutic intervention. Earlier mutagenesis studies indicated that *Acsl4* is an essential gene for the execution of ferroptosis induced by inhibition or degradation of glutathione peroxidase 4 (GPX4), an enzyme that uses glutathione to detoxify lipid peroxides [10]. Therefore, we disrupted this gene using CRISPR (clustered regularly interspaced short palindromic repeat)/Cas9-based genome-editing technology [18]. Knockout of *Acsl4* in the otherwise ferroptosis-sensitive murine NIH3T3 and human HT-1080 cells was verified by loss of protein expression (Fig. 1a and Supplementary Material, Fig. S1a). To further assess the relative roles of ferroptosis and necroptosis, we disrupted *Mlkl* by the same technique in murine NIH3T3 as well as human HT-29 cells (Fig. 1b and Supplementary Material, Fig. S1b). The latter cell lines were selected partly because HT-1080 cells do not express RIPK3 and therefore are *per se* not sensitive for RIPK3-MLKL-dependent necroptosis. Like the murine fibroblastoma L929 cells, HT-29 cells express wildtype, rather than oncogenic HRAS, which renders them less sensitive to erastin-induced and RSL3-induced ferroptotic cell death [19]. However, NIH3T3 cells are susceptible to death-receptor-ligand-mediated necroptosis involving MLKL as well as erastin-induced and RSL3-induced ferroptosis (Fig. 1c and Supplementary Material, Fig. S1c). Therefore, we focused our subsequent in vitro studies on this cell line, in which we could examine both necroptosis and ferroptosis. As expected, *Acsl4*-knockout NIH3T3 cells were protected from erastin-induced as well as RSL3-induced ferroptosis, but underwent necroptosis after treatment with a combination of the death receptor ligand TNFα and the pan-caspase inhibitor zVAD-fmk (TZ) (Fig. 1d and Supplementary Material, Fig. S1d). Reciprocally, *Mlkl*-knockout NIH3T3 cells were protected from TZ-induced necroptosis, but underwent ferroptotic cell death after incubation with the small molecules, erastin or RSL3 (Fig. 1e and Supplementary Material, Fig. S1e). To best illustrate these findings, we have presented a representative FACS dot plot from each experiment in Fig. 1c–e, in addition to summary data representing the mean and standard deviation of four independent experiments. Furthermore, results obtained by flow cytometry quantification of cell death were verified by measuring loss of plasma membrane integrity using LDH release assays (Supplementary Material, Fig. S1f). We verified expression of ACSL4 in genetically unmodified parental HT-29 and L929 cells by Western blotting, thus ruling out the possibility that loss of ACSL4 expression might be a protective mechanism of cancer cells to avoid their own demise by ferroptosis (Supplementary Material, Fig. S1g). The fact that *Acsl4*-edited cells still died by necroptosis and *MLKL*-edited cells by ferroptosis indicated that both signaling pathways operate independently from one another, although they can contribute in a combined fashion to mediate regulated cell death processes [12].

### Necroptosis and ferroptosis, two pathways of regulated necrosis, are alternative

**Fig. 2:**
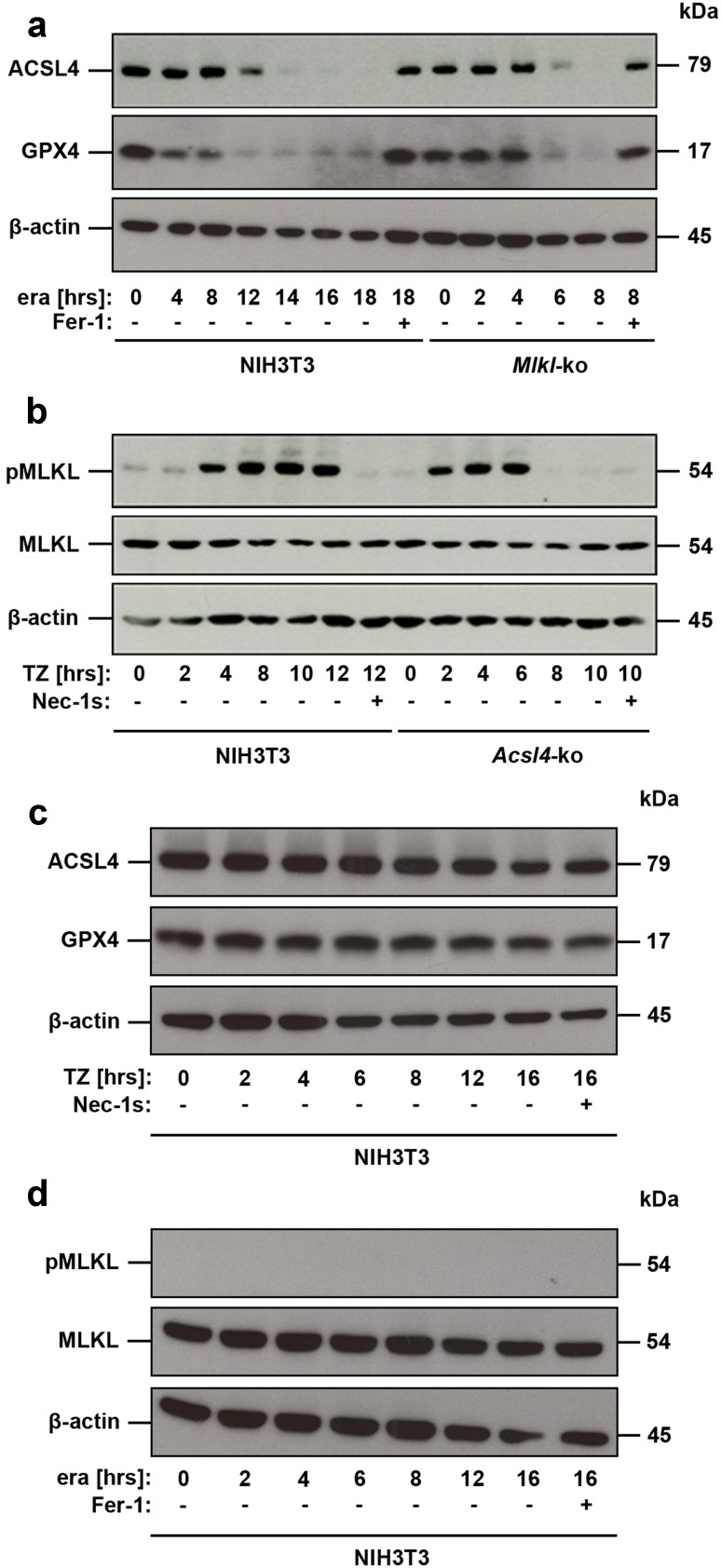
Loss of ferroptosis or necroptosis signaling sensitizes cells to the alternate pathway. **a** Genetically unmodified NIH3T3 and *Mlkl*-edited NIH3T3 cells were left untreated or were stimulated for different time points with 10 µM erastin (era) in the presence or absence of 1 µM ferrostatin (Fer-1), as indicated. Equal amounts of protein (20 µg/lane) were resolved by SDS/PAGE and expression of ACSL4 (*M*_r_ = 79.0 kDa) was detected by Western blotting. Notably, different stimulation periods are shown to most clearly illustrate the differences observed between parental and edited cells (see axis labeling). The blot was stripped and re-probed first with an antibody against GPX4 (*M*_r_ = 17.0 kDa, predicted molecular weight = 22.0 kDa) and thereafter with an antibody against β-actin as loading control. **b** Genetically unmodified NIH3T3 and *Acsl4*-edited NIH3T3 cells were left untreated or were stimulated for different time points with 100 ng/ml TNFα + 25 µM zVAD (TZ) in the presence or absence of 50 µM necrostatin-1_s_ (Nec-1_s_), as indicated. Equal amounts of protein (20 µg/lane) were resolved by SDS/PAGE and expression of phospho-MLKL (*M*_r_ = 54.0 kDa) was detected by Western blotting. Notably, different stimulation periods are shown to most clearly illustrate the differences observed between parental and edited cells (see axis labeling). The blot was stripped and re-probed first with an antibody against whole MLKL (*M*_r_ = 54.0 kDa) and thereafter with an antibody against β-actin as loading control. **c** Genetically unmodified NIH3T3 cells were left untreated or were stimulated for different time points with 100 ng/ml TNFα + 25 µM zVAD (TZ), as indicated. Equal amounts of protein (20 µg/lane) were resolved by SDS/PAGE and expression of ACSL4 was detected by Western blotting. The blot was stripped and re-probed first with an antibody against GPX4 (*M*_r_ = 17.0 kDa, predicted molecular weight = 22.0 kDa) and thereafter with an antibody against β-actin as loading control. **d** Genetically unmodified NIH3T3 cells were left untreated or were stimulated for different time points with 10 µM erastin (era), as indicated. Equal amounts of protein (20 µg/lane) were resolved by SDS/PAGE and phosphorylated MLKL was detected by Western blotting. The blot was stripped and re-probed first with an antibody against whole MLKL and thereafter with an antibody against β-actin as loading control

The data above suggest that *Acsl4* deletion, mutation or expression may aid in predicting sensitivity to ferroptotic cell death, thereby serving as a predictive biomarker. This finding led us to hypothesize that ACSL4 levels could potentially also serve as a pharmacodynamic marker of the execution of ferroptosis and thus overcome the dearth of suitable markers in this field for monitoring pathway activation. We examined regulation of ACSL4 at protein level in genetically unmodified NIH3T3 undergoing ferroptosis, revealing that ACSL4 levels decreased in a time-dependent manner. Notably, ACSL4 protein was no longer detectable 14 h post induction of ferroptosis (Fig. 2a). Pretreatment of these cells with ferrostatin-1 (Fer-1), an arylalkylamine that was identified as one of the first chemical inhibitors of ferroptosis, which has been suggested to act by preventing oxidative damage to membrane lipids [7], blocked ACSL4 degradation completely (Fig. 2a). These data validate the integral role of ACSL4 in ferroptosis, and its suitability as a marker of this regulated cell death modality.

While much remains to be learned, recent studies have identified ferroptosis regulators and the molecular mechanism underlying this mode of cell death [20]. One key component of the ferroptosis pathway is GPX4, which can inhibit ferroptosis by reducing lipid peroxidation [14]: a process negated by its degradation following erastin treatment [21]. Therefore, we examined a possible link between ACSL4 and GPX4 depletion in our cellular models by Western blot analysis (Fig. 2a). These studies revealed simultaneous degradation of both ACSL4 and GPX4 after stimulation with erastin, suggesting a common feedback pathway affecting both proteins. Unexpectedly, we detected a significant acceleration in degradation of ACSL4 in necroptosis-incompetent *Mlkl*-edited NIH3T3 cells relative to the parental cells, with detectable protein levels observed only up to 6 h after stimulation rather than 12 h in the parental counterparts (Fig. 2a), Such an accelerated ferroptotic program was echoed in the degradation of GPX4 in these necroptosis-incompetent cells (Fig. 2a), in spite of identical test conditions between parental and *Mlkl*-edited NIH3T3 cells.

Interestingly, we observed the reciprocal phenomenon in the *Acsl4*-edited NIH3T3 for activated MLKL (pMLKL), where phosphorylated MLKL was maximally detected 6 h after necroptotic stimulation compared to 12 h in the unedited counterparts (Fig. 2b). Correspondingly, the *Acsl4*-edited NIH3T3 cells were more susceptible to necroptosis compared to the unmodified NIH3T3 cells. It should be noted that different stimulation periods are shown (as indicated in the Figure legend) to most clearly illustrate the differences observed between parental and edited cells (Fig. 2).

To exclude the possibility that non-specific effects of stimuli used to induce ferroptosis or necroptosis may contribute to our observations, we examined ACSL4 and GPX4 expression levels in unmodified NIH3T3 cells in a time-dependent manner after induction of TZ-mediated necroptosis (Fig. 2c) and, complementarily, MLKL expression in unmodified NIH3T3 cells after induction of ferroptosis (Fig. 2d). These time courses (up to 16 h) indicate that induction of necroptosis (confirmed simultaneously by FACS analysis, data not shown) does not alter ACSL4 and GPX4 expression in unmodified NIH3T3 cells. Analogously, induction of ferroptosis by erastin-stimulation in non-edited NIH3T3 cells (confirmed simultaneously by FACS analysis, data not shown) did not alter MLKL expression, nor induce MLKL phosphorylation, in these cells over the entire time course (Fig. 2d). These data eliminate the possibility that degradation of these core effector molecules following treatment with necroptotic or ferroptotic stimuli might account for the observed alternative between death modalities.

**Fig. 3:**
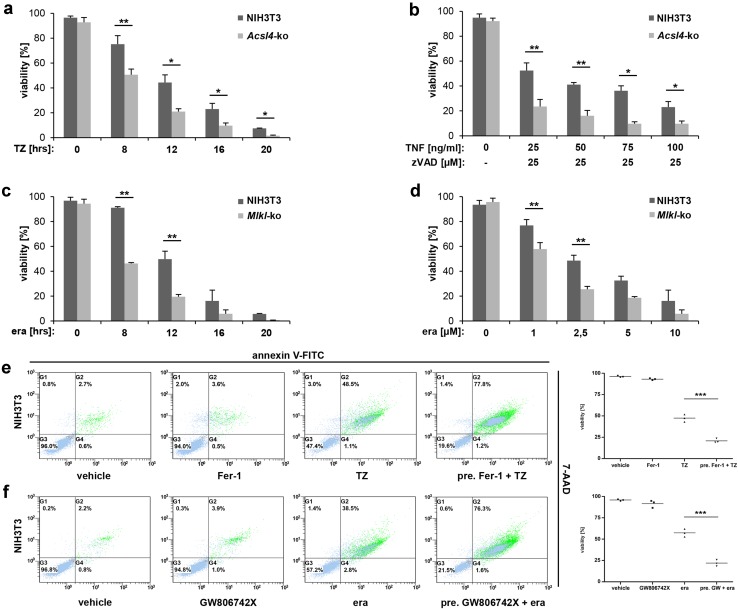
Alternative of necroptotic and ferroptotic pathways was seen across a range of time points and stimuli concentrations. **a**, **b**, **e** Detection of necroptosis in parental and *Acsl4*-edited NIH3T3 cells, respectively. **a** The cells were treated at the time indicated with 100 ng/ml TNFα + 25 µM zVAD (TZ) or **b** for 16 h with constant 25 µM zVAD and different (indicated) concentration of TNFα. Mean ± SD is shown for three independent experiments. **e** FACS analysis of parental NIH3T3 cells that were pretreated as indicated for 30 min with 1 µM ferrostatin (Fer-1). Necroptosis was induced thereafter for 12 h by the addition of 100 ng/ml TNFα + 25 µM zVAD (TZ). Depicted is one of three independent experiments. **c**, **d**, **f** Detection of ferroptosis in parental and *Mlkl*-edited NIH3T3 cells, respectively. **c** Cells were treated for indicated time at 37 °C with 10 µM erastin (era) or **d** for 16 h with different (indicated) concentration of erastin. Mean ± SD is shown for three independent experiments. **f** FACS analysis of parental NIH3T3 cells that were pretreated as indicated for 30 min with 2.5 µM GW806742X. Ferroptosis was induced thereafter for 12 h by the addition of 10 µM erastin (era). **e**, **f** FACS dot plots of one representative experiment are shown, with adjacent *box plots* presenting the mean and standard deviation of three independent experiments

These observations prompted us to investigate the interdependency of necroptotic and ferroptotic pathways in real time at the cellular level in a time- and concentration-dependent manner. By initially using a single TZ-concentration, we detected an increased time-dependent sensitivity to necroptosis in the *Acsl4*-knockout cells that was not observed in unmodified NIH3T3 cells (Fig. 3a). The hypersensitization of the *Acsl4*-knockout cells to necroptotic cell death was also clear at a 16 h time point across a range of TNFα concentrations (25 - 100 ng/ml, Fig. 3b). These data support that loss of *Acsl4* predisposed cells to necroptosis, and correlated with elevated MLKL activation. We substantiated this hypothesis by pretreatment of the parental NIH3T3 cells with the ferroptosis inhibitor, ferrostatin-1 (Fer-1). As expected, Fer-1 pretreated fibroblasts were protected from ferroptotic stimuli, but notably were sensitized to TZ-induced necroptosis relative to non-Fer-1 pretreated NIH3T3 cells (Fig. 3e). The interdependence of necroptosis and ferroptosis was clearly demonstrated using the *Mlkl*-knockout NIH3T3 cell line, where loss of MLKL enhanced sensitivity to ferroptosis stimuli (Fig. 3c, d, f). Initially, we induced ferroptosis in *Mlkl*-knockout cells with 10 µM erastin, leading to clear time-dependent increases in ferroptosis (Fig. 3c) that was also evident at erastin concentrations as low as 1 µM (Fig. 3d). Further support for the idea that RCD signaling pathways are intertwined and interact with each other arose from studies of the parental NIH3T3 cells pretreated with the small molecule GW806742X [22] which is described as an inhibitor of the protein kinase VEGFR2, but also functions as a potent binder of murine MLKL [5] and RIPK1 [23]. GW806742X, also known as “Compound 1” was recently described to target the pseudokinase domain of MLKL, whereas inhibition of VEGFR2 does not play a part in the inhibition of necroptosis [5]. Therefore, GW806742X is currently the best available drug for inhibition of murine MLKL, because another compound, necrosulfonamide, only targets the human ortholog.

Consistent with the outcomes of genetic knockout experiments, NIH3T3 cells pretreated with GW806742X to block MLKL-dependent necroptosis exhibited increased sensitivity to erastin-induced ferroptosis (Fig. 3f; FACS dot plot shown for a representative experiment alongside mean and standard deviation of three independent experiments). Interestingly, we did not observe this increased sensitivity to erastin-mediated ferroptosis following pretreatment of the parental NIH3T3 cells with a stable analog of the necroptosis inhibitor necrostatin-1 (Nec-1_s_), which targets RIPK1 [24], and the RIPK3 inhibitors, GSK’872 and dabrafenib [25, 26] (Supplementary Material, Fig. S2a), which suggests that the mechanism of cross-pathway alternative is downstream of RIPK3. To further examine the manner by which ferroptosis is activated, we also assessed the lipid ROS production over time by flow cytometry using BODIPY^®^ (581/591) C11. Once again, our findings with *Mlkl*-knockout NIH3T3 cells support the notion that loss of *Mlkl* sensitizes cells to ferroptotic death (Supplementary Material, Fig. S2b).

### Necroptosis and ferroptosis act independently in numerous pathologies driven by cell death, but are intertwined in murine renal IRI

**Fig. 4:**
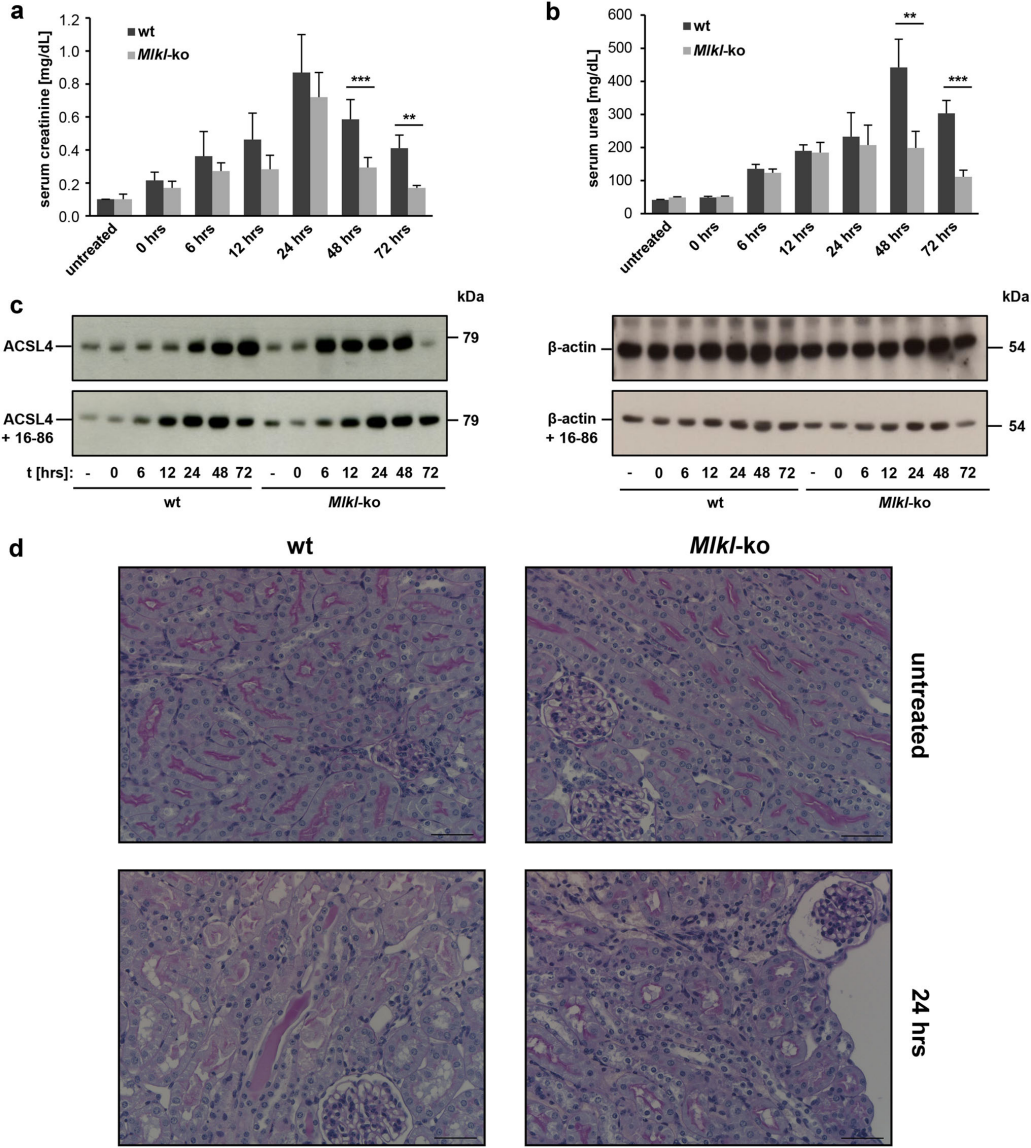
Necroptosis and ferroptosis are intertwined in murine IRI. Male C57BL/6 wildtype mice and *Mlkl*-knockout mice underwent 35 min of bilateral pedicle clamping (ischemia) followed by different times of reperfusion. **a** Serum creatinine and **b** serum urea concentrations were determined, respectively, after sacrificing the mice at the indicated reperfusion time points. **c** *left* Expression levels of ACSL4 (79.0 kDa) in whole-kidney lysates taken from wildtype or *Mlkl*-knockout mice during the time course of IRI. A concomitant treatment of the animals for the first 24 h after the onset of reperfusion with the ferrostatin-derivative 16-86 in this setting is illustrated in the lower part. **c** *right* Thereafter, the blots were stripped and re-probed with an antibody against β-actin as loading control. **d** Representative histological sections of murine kidneys (contralateral to **c**) after staining with periodic acid-Schiff (PAS) show less severe tubular damage in *Mlkl*-knockout mice compared to C57BL/6 wildtype mice. Depicted are representative enlarged sections of the untreated groups and 24 h IRI values, respectively (*n* = 4 animals for each group and time point, *scale bars* 50 µm)

Our previous in vivo studies demonstrated that two independent regulated necrosis (RN) pathways—necroptosis and ferroptosis—contribute to the same IRI process [12, 16], but it remains of outstanding interest whether these individual RN pathways are functionally interconnected. Because the in vitro data described above using the *Acsl4*- and *Mlkl*-edited NIH3T3 cells provide support for inter-pathway alternative, it was thus of interest to examine whether this impacts pathogenesis in relevant in vivo disease models. Due to the partial preweaning lethality of constitutive *Acsl4*-knockout mice (only females are formally homozygous, since the gene of interest is located on X-chromosome), we subjected *Mlkl*-knockout mice maintained on a C57BL/6 background, and C57BL/6 wildtype counterparts, to acute ischemic reperfusion injury (IRI). As noted previously [15], the *Mlkl*-knockout mice did not show any overt phenotypic abnormalities under non-challenged conditions, were fertile and bred as homozygotes. To track the expression level of ACSL4 in a time course of cell death, mice underwent 35 min of bilateral pedicle clamping followed by different times (up to 72 h) of reperfusion. Wildtype mice exhibited elevated serum concentrations of creatinine (Fig. 4a) and urea (Fig. 4b) up to 24 and 48 h of reperfusion, respectively, indicative of compromised kidney function. By contrast, the *Mlkl*-knockout mice exhibited slight protection in this model, which was most evident, relative to identically-handled wildtype C57BL/6 counterparts, beyond the 24 h time point. Interestingly, this slight protection of *Mlkl*-knockout mice correlates with elevated expression of ACSL4 within the first 24 h of reperfusion, whereas ACSL4 expression was only markedly elevated beyond 24 h in wildtype mice (Fig. 4c top and repeated in Supplementary Material, Fig. S3a). In contrast, treatment of animals with the ferrostatin-derivative, 16–86 [12] (Fig. 4c bottom), reduced expression of ACSL4 in *Mlkl*-deficient mice to a level comparable to those observed in wildtype counterparts post-reperfusion with similar expression kinetics. The reduction of ACSL4 expression to wildtype levels by concomitant treatment with 16–86, and the aforementioned in vitro data (Figs. 2, 3), provide support for the idea that elevated ACSL4 expression in mice will be accompanied with an increased ratio of ferroptotic-mediated cell death in this model. Notwithstanding this, the pharmacologically-induced reduction—albeit not elimination—of ACSL4 expression in *Mlkl*-knockout mice at the outset of reperfusion did not correspond to an improved outcome following IRI at the 72 h post-reperfusion time point compared to vehicle-treated *Mlkl*-knockout mice alone, most likely reflecting the in vivo lability of 16–86. In contrast, in wildtype mice, we observed a time-dependent elevation in MLKL protein expression post-reperfusion, which is consistent with necroptosis contributing to the pathology of IRI (Supplementary Material, Fig. S3b). We also examined MLKL phosphorylation at S345, an event known to promote MLKL-mediated cell death [15], as a candidate biomarker for necroptotic cell death in primary cells and tissues. In contrast to lysates obtained from transformed cell lines treated with necroptotic stimuli (e.g. by addition of TZ; Supplementary Material, Fig. S3e), and other reported in vivo studies [27, 28], we were not able to detect authentic phosphorylated MLKL (pMLKL) in any kind of primary mammalian material, either by performing Western blot analysis (Supplementary Material, Fig. S3c) nor immunostaining (Supplementary Material, Fig. S4c). While we do not have a simple explanation for the differences in our findings and those reported previously, it is possible that variations in head-to-head controls (wildtype vs. *Mlkl*-knockouts) (Supplementary Material, Fig. S3, S4) and loading controls (Supplementary Material, Fig. S3d), as well differences in experimental protocols, may have led to different results or interpretations. As described above, our data show a strong correlation between increased ACSL4 expression at the onset of reperfusion in whole cell lysates of the kidneys from *Mlkl*-knockout mice, as detected by Western blotting (Fig. 4c top), and the corresponding histology from the contralateral kidney (Supplementary Material, Fig. S4a). Similarly, we observed a time-dependent increase in MLKL protein expression in wildtype mice following the onset of reperfusion (Supplementary Material, Fig. S3b), which was reflected in the analogous histopathological staining (Supplementary Material, Fig. S4b). Notably, reliable pMLKL detection in wildtype mice in this setting was not possible by immunostaining using the aforementioned commercial antibodies, with only non-specific staining evident from parallel, head-to-head examination of *Mlkl*-knockout controls (Supplementary Material, Fig. S4c). Although we tested a variety of different conditions for tissue preparation, antigen retrieval or protein blotting, in our hands, none of these conditions proved to be suitable for the detection of endogenous pMLKL in primary tissues. However, we cannot exclude the possibility that phosphorylation of MLKL at sites other than the activation loop [29], which is the target of the pMLKL antibody, contribute to this mode of cell death. Further studies will be required to address this in detail. Although MLKL is thought to be the principal substrate of RIPK3 in necroptosis signaling, the molecular determinants acting downstream of MLKL remain unclear. As a result, it remains of ongoing interest whether activated MLKL is the terminal executioner of necroptotic cell death, whether additional proteins are required to augment or activate its killer function, and/or whether other proteins act downstream of MLKL to induce cell death. It is therefore foreseeable that as we learn more about the necroptosis pathway, future studies may uncover additional biomarkers downstream of activated MLKL.

While commercially-available reagents were not suitable for pMLKL detection, histological analysis over the entire observation period revealed diminished kidney damage after renal IRI in *Mlkl*-knockout (*Mlkl*-ko) mice, but not wildtype counterparts (wt), beyond 24 h post-ischemia. At 24 h reperfusion, wt mice showed extensive necrosis of tubular epithelia with detachment, in addition to loss of brush border and polarity, following IR (Fig. 4d, lower left). These changes are only focal and pronounced in the *Mlkl*-ko mice after IR (Fig. 4d, lower right). The tubular damage is potentially reversible, but would initiate an inflammatory response and negatively influence long term kidney function. Focal interstitial leukocyte infiltrates can be seen in the wt animals. No tubular damage was observed in each of the untreated wt and *Mlkl*-ko animals.

### Increased expression of ACSL4 in human kidney transplants indicates a role for ferroptotic cell death in vivo

**Fig. 5:**
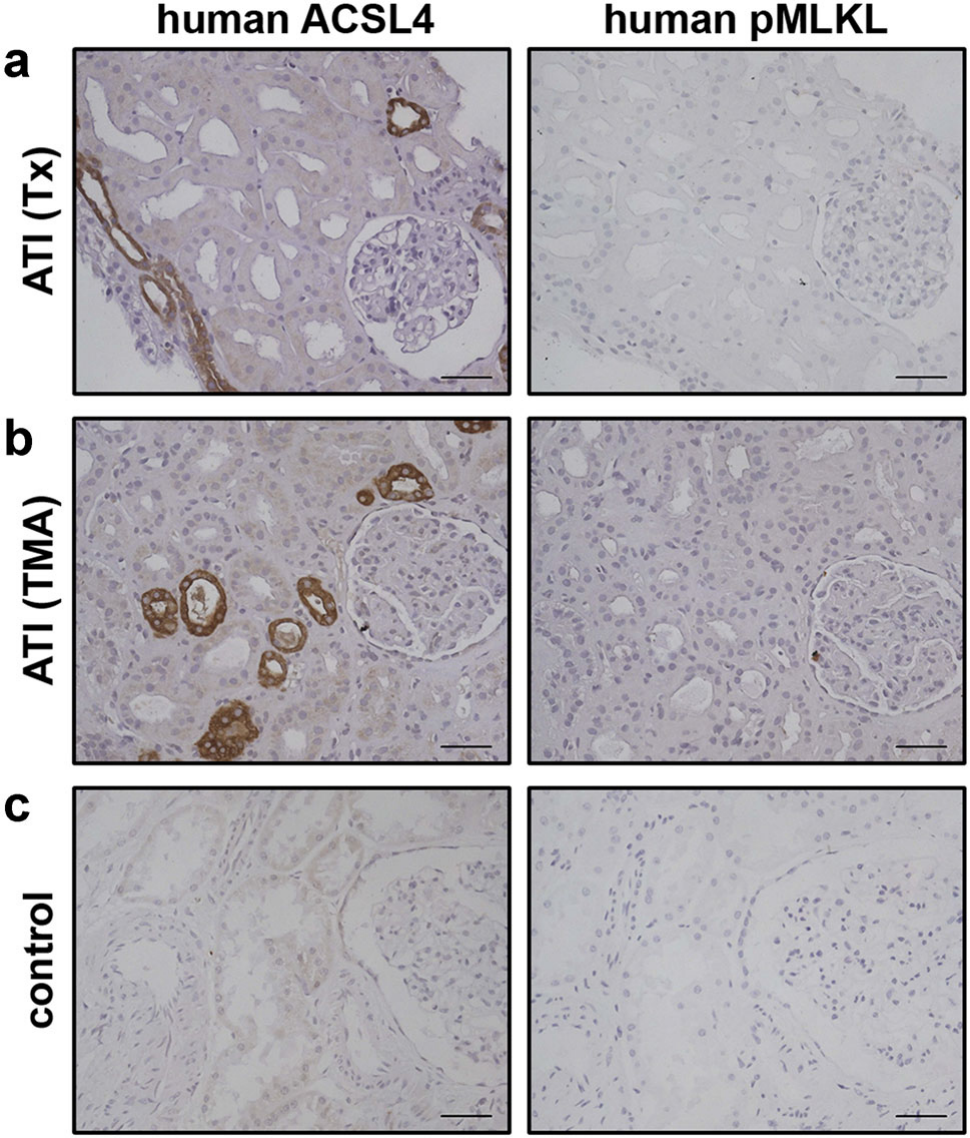
Increased expression of ACSL4 in human kidney biopsies indicates a role for ferroptotic cell death in vivo. Human kidney biopsies were stained for ACSL4 and phospho-MLKL as indicated. **a** Positive tubuli can be seen in acute tubular injury 7 days post-transplantation (transplanted kidney (Tx) from a female patient showing moderate tubular injury) and **b** severe acute tubular damage (male patient suffering from thrombotic microangiopathy (TMA) due to atypical hemolytic-uremic syndrome). Notable, increased expression of ACSL4 verifies an important role of ferroptosis in the complex pathology of acute tubular injury (ATI) and distinguished for the first time ACSL4 as a biomarker of pathological ferroptosis. **c** Normal renal parenchyma samples taken from a tumor nephrectomy of renal cell carcinoma served as controls (*scale bars* 60 µm). The biopsies presented in this figure are representative of a total of ten human biopsies (*n* = 10)

Expression and/or activation of RIPK1, RIPK3 and MLKL have been established as RCD markers in patient biopsies (summarized in [30]) and possible therapeutic targets in necroptosis-driven pathologies. Despite its pathophysiological importance, similar types of markers have not been identified so far for ferroptotic cell death. Consequently, we explored whether increased expression of ACSL4 could serve as a *bona fide* RCD marker in human kidney biopsies from patients with acute tubular injury (ATI), and in so-doing, verify a role of ferroptosis in this complex pathology for the first time. As demonstrated in Fig. 5, we detected robust immunostaining of ACSL4 in human ATI seven days post-transplantation (Fig. 5a), as well as in severe thrombotic microangiopathy of native kidney (Fig. 5b). Normal renal parenchyma samples taken from a tumor nephrectomy of renal cell carcinoma served as controls (Fig. 5c). Although we suggest that necroptosis can occur in parallel in the same transplanted organ sections, available reagents did not permit us to detect phosphorylated MLKL in these biopsies. Nevertheless, these results highlight for the first time ACSL4 as a biomarker of pathological ferroptosis. While the in vitro data reveal an unexpected alternative between the ferroptosis and necroptosis cell death pathways, further investigation of their relationship in pathological settings will rely on the development of more sensitive phospho-specific reagents.

## Discussion

The ability to compare the contributions of different cell death pathways and analyze their cross-talk in pathophysiological processes would be beneficial for improving our understanding of the mechanisms governing regulated cell death and their roles in complex diseases. However, clinically-useful predictive biomarkers that reflect the impact of ongoing regulated necrosis are lacking so far [31]. One reason for this might be that most of the available antibodies that recognize specific phosphorylation sites on key proteins which mediate the different forms of regulated necrosis are only suitable for immunoblotting [32]. Regardless, diverse pathologies associated with regulated necrotic cell death routines, such as myocardial infarction, stroke, sepsis and cancer [23], as well as solid organ transplantation indicate the increasing clinical importance of such biomarkers for use in monitoring, diagnosis and also drug development.

It has been known for several years that repression of necroptotic pathways by apoptotic regulators, such as FADD and caspase-8, is essential for proper mammalian development and survival [33, 34]. Otherwise, depending on cell type, genetic background and microenvironmental conditions, cell fate may be shifted from apoptosis to necrosis [35, 36]. Regulated necrosis includes multiple cell death modalities such as necroptosis, mitochondrial permeability transition pore (MPTP)-mediated necrosis, ferroptosis, parthanatos, and pyroptosis, but not apoptosis [37]. Our earlier studies revealed that the mechanistically distinct regulated necrotic pathways, necroptosis and ferroptosis, can contribute to the pathogenesis of a single disease, such as acute kidney injury [12, 16]. Results of further studies even suggest that ferroptosis could be a common denominator in IRI, independent of tissue origin [8, 12]. At the molecular level, dysregulation of lipid metabolism was recently associated with ferroptosis [38], but the signals that link lipid modifying proteins to the process of ferroptosis have not been identified so far.

By using the CRISPR/Cas9-based genome-editing technology we illustrate that the ablation of *Acsl4* in different cell lines appears dispensable for proliferation in these cells but confers resistance to ferroptosis, possibly via the decrease of PUFAs in specific phospholipids. To exclude possible off-target effects we generated and tested three different guide RNAs (see Materials and methods) for *Acsl4* and *Mlkl* in both mouse and human cell lines, and observed congruent outcomes in each case (data not shown). Kinetic studies in different cultured cell lines, and models of acute renal failure in mice and humans, demonstrated that ACSL4 was initially upregulated in non-fatal ferroptosis, thereby uncovering this molecule as the first reliable protein biomarker for the detection of this cell death modality. In parallel to our submission, another group confirmed our in vitro results showing that ACSL4 contributes to ferroptosis in HepG2 and HL60 cells [13]. Our finding was echoed in cellular studies in vitro, where initiation of ferroptotic cell death correlated with progressive ACSL4 protein level reduction. It has been shown previously that increased GPX4 degradation contributes to ferroptotic cancer cell death [38]. Here, we observed that ACSL4 and GPX4 were degraded in synchrony during ferroptotic cell death, although the underlying mechanism remains a matter of ongoing interest. Chemical inhibitors of ferroptosis, like Fer-1, suppressed these protein expression kinetics (Fig. 2a). These data are consistent with the idea that ACSL4 expression level correlates with the induction and progress of ferroptotic cell death, and monitoring ACSL4 protein level would be suitable for diagnostic purposes.

Additionally, comparative studies with *Mlkl*-knockout cells and mice demonstrated for the first time that the presumed mechanistically-distinct and independent modes of regulated necrosis, necroptosis and ferroptosis, are not only interconnected, but also are alternative to one another in a setting where both contribute to cell death and, one has been compromised. The mechanism underlying this phenomenon remains a matter to be addressed in the future, but it is possible that membrane lipid composition represents a point of alternative between ferroptosis and necroptosis, where MLKL drives basal resistance to ferroptosis through depleting PUFAs, and ACSL4 drives basal resistance to necroptosis by making the membrane less amenable to MLKL-driven membrane permeabilization. Our data support the crosstalk occurring at the level of MLKL or via as-yet-unidentified downstream effectors, because pharmacological inhibition of the necrosome component kinases, RIPK1 and RIPK3, did not impact ferroptosis in our cellular studies (see Supplementary Material, Fig. S2a). Nevertheless, further studies are needed to confirm whether unchanged ACSL4 expression contributes to ferroptosis resistance in other tumors and diseases. Interestingly, interpathway crosstalk has emerged as an important regulatory mechanism in cell death. For example, the autophagy machinery can serve as a scaffold to control the switch between necroptosis and apoptosis in the context of *Map3k7* loss [39], and MLKL was recently shown to activate the inflammasome protein, NLRP3 [40].

Activation of ACSL4 in the course of ferroptosis represents an important but poorly understood phenomenon, as do the details of how loss of *Acsl4* confers protection against ferroptosis. In cells undergoing ferroptosis, PUFAs such as arachidonic acid (AA) are significantly depleted [9, 41]. *Acsl4* encode an enzyme that is involved in the insertion of AA into membrane phospholipids. These PUFAs might change the biophysical properties of cell membranes, such as lipid rafts. Lipids are emerging as key components of several non-apoptotic RCD pathways [42]. Therefore, it is conceivable that the initiation and execution of ferroptosis can only proceed when highly oxidizable PUFAs such as AA are present in the membrane. The identification of ACSL4 loss as a key mediator of resistance to ferroptosis supports this mechanism.

In a similar vein, the data presented here demonstrate that alternative signal transduction pathways of regulated necrosis act in opposition in pathophysiological processes of acute kidney failure and each of them can contribute to the total organ damage. These findings need to be taken into consideration in the conception and development of pharmacological strategies for therapeutic intervention where therapeutic targeting of both ferroptosis and necroptosis may be necessary to negate complex diseases like AKI. To explore this idea further, we attempted to model dual inhibition of ferroptosis and necroptosis in murine IRI by administration of the ferroptosis inhibitor, 16–86, in necroptosis-insensitive *Mlkl*-knockout mice. Surprisingly, although 16–86 treatment reduced ACSL4 levels in *Mlkl*-knockout mice, this was not reflected in protection from IRI. This finding is consistent with the inherent instability of 16–86 in vivo over the time frame required for the IRI model, which limits its effectiveness in in vivo settings at this stage. Additionally, because we focused on the 24 h reperfusion time point, when kidney damage had begun and was accompanied by a clear window between ACSL4 levels in *Mlkl*-knockout and wt mice, we stopped the therapy with 16–86 24 h after the onset of reperfusion. Termination of the experiment at this time point could explain why these mice did not exhibit significant protection relative to vehicle-treated *Mlkl*-knockout mice. Furthermore, it is worth mentioning that detection and diagnosis of AKI relies usually on changes in serum creatinine and urea. Individually, they remain the longstanding parameters used to test renal function, even though are known to be imperfect because they do not reflect genuine injury or real-time changes in kidney function [43]. Accumulation and concomitant increase of serum creatinine concentration lags far behind renal injury, which causes deterioration of glomerular filtration rate. Thus, substantial rises in serum creatinine are often not witnessed until 24–72 h after the initial insult to the kidney. Meanwhile, it is presumed that *de novo* synthesized proteins are more suitable for early detection of AKI. ACSL4 is one such protein, because it is expressed early after injury and therefore has value in predicting AKI in clinical settings, such as after kidney transplant.

More detailed analyses of the therapeutic potential of pharmacological targeting of both necroptosis and ferroptosis in complex disease models will depend on future development of less labile ferroptosis inhibitors. The data presented herein suggest that combination therapy using anti-necroptosis and anti-ferroptosis compounds, or small molecules (e.g. chimera) that simultaneously inhibit both pathways, with concomitant enhancements in in vivo efficacy, may be beneficial for the prevention of IRI; of course, it may be necessary to additionally disable other pathways at the same time. While these ideas represent an exciting therapeutic possibility, clinical translation of these findings will rely on future development of novel inhibitors of regulated necrosis with improved potency and plasma stability suitable for efficient application in diseases.

## Electronic supplementary material

Below is the link to the electronic supplementary material.

Supplementary material 1 (TIFF 932 kb)

Supplementary material 2 (TIFF 573 kb)

Supplementary material 3 (TIFF 397 kb)

Supplementary material 4 (TIFF 7117 kb)

Supplementary material 5 (DOCX 52 kb)
