# Supplementary figures and images for: Necroptosis and ferroptosis are alternative cell death pathways that operate in acute kidney failure
Source: Cell Mol Life Sci. 2017 May 27;74(19):3631–45. doi: 10.1007/s00018-017-2547-4 (PMC5589788; doi:10.1007/s00018-017-2547-4)

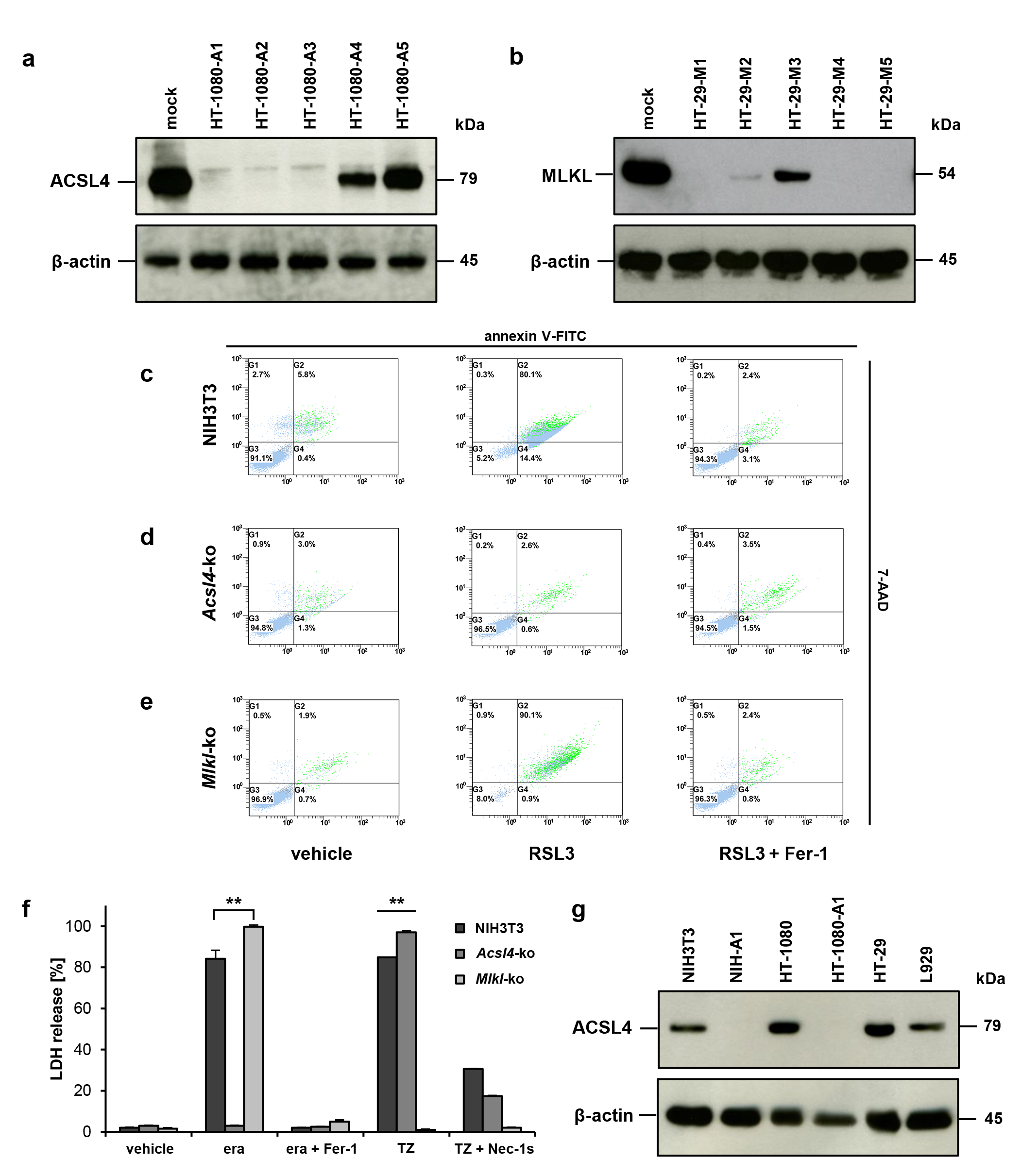

Supplement: Supplementary file 1 — Supplementary material 1 (TIFF 932 kb) [file 18_2017_2547_MOESM1_ESM.tif]

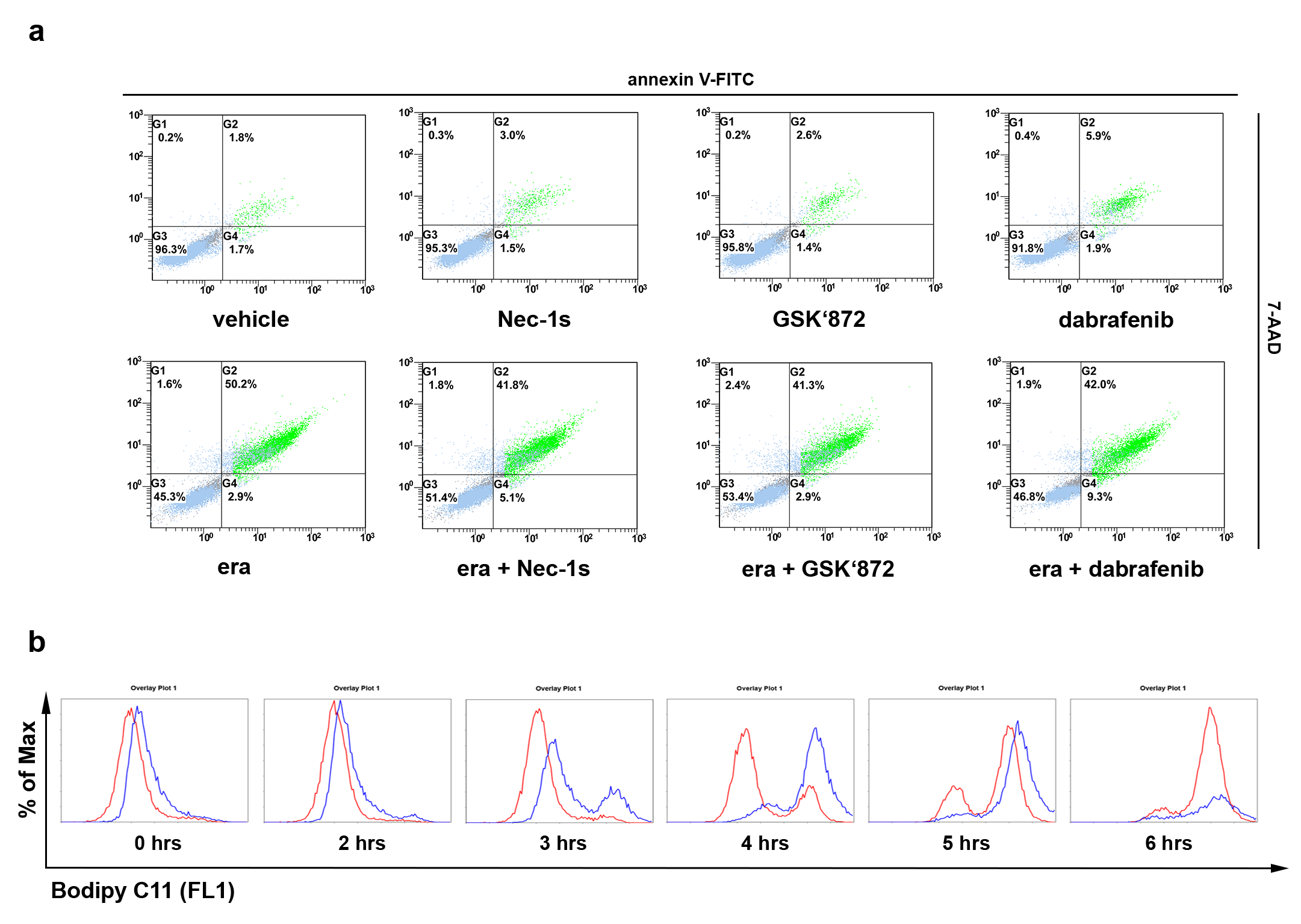

Supplement: Supplementary file 2 — Supplementary material 2 (TIFF 573 kb) [file 18_2017_2547_MOESM2_ESM.tif]

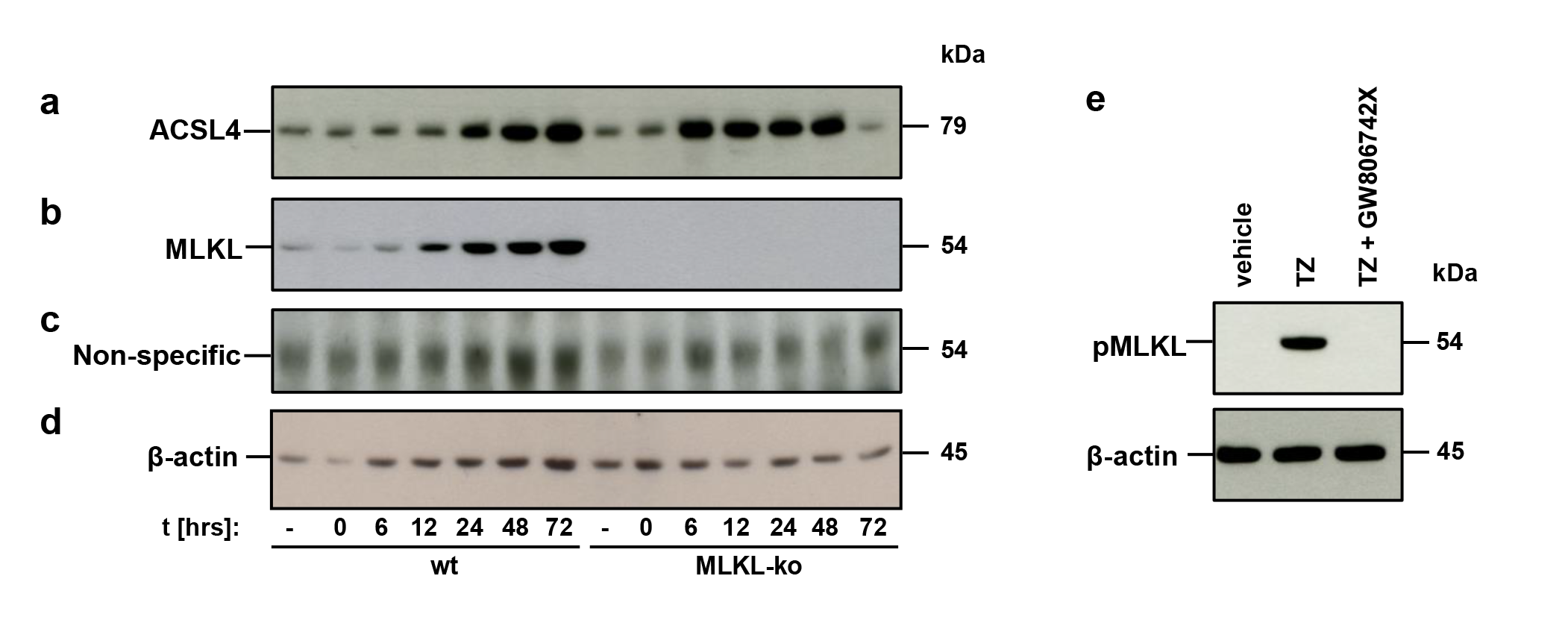

Supplement: Supplementary file 3 — Supplementary material 3 (TIFF 397 kb) [file 18_2017_2547_MOESM3_ESM.tif]

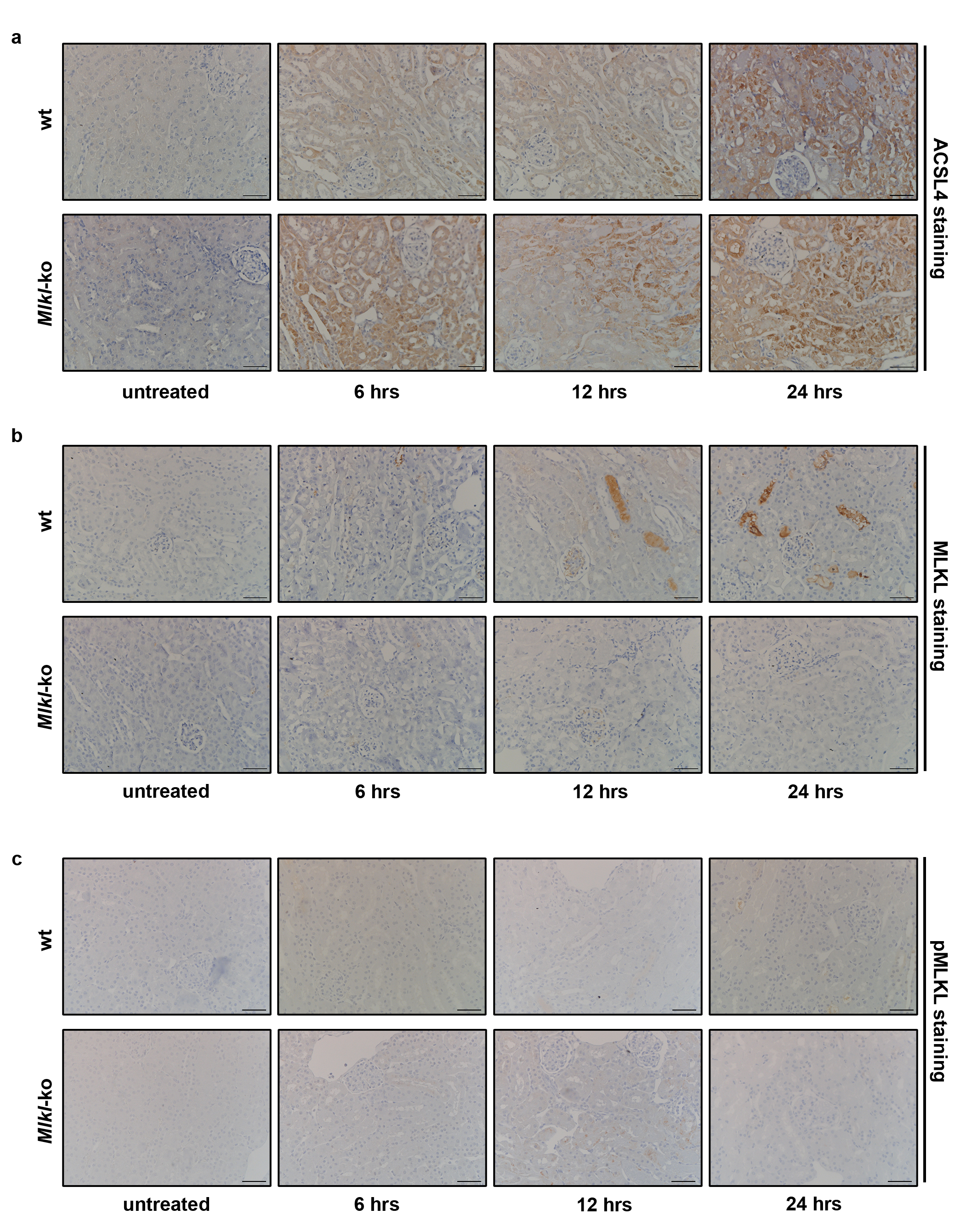

Supplement: Supplementary file 4 — Supplementary material 4 (TIFF 7117 kb) [file 18_2017_2547_MOESM4_ESM.tif]
