# Supplementary material for: Necroptosis and ferroptosis are alternative cell death pathways that operate in acute kidney failure
Source: Cell Mol Life Sci. 2017 May 27;74(19):3631–45. doi: 10.1007/s00018-017-2547-4 (PMC5589788; doi:10.1007/s00018-017-2547-4)
Supplement: Supplementary file 5 — Supplementary material 5 (DOCX 52 kb) [file 18_2017_2547_MOESM5_ESM.docx]

**Supplementary material**

**Necroptosis and ferroptosis are alternative cell death pathways that operate in acute kidney failure**

Tammo Müller^1^, Christin Dewitz^1^, Jessica Schmitz^2^, Anna Sophia Schröder^1^, Jan Hinrich Bräsen^2^, Brent R. Stockwell^3^, James M. Murphy^4, 5^, Ulrich Kunzendorf^1^ and Stefan Krautwald*^1^

^1^Department of Nephrology and Hypertension, University Hospital Schleswig-Holstein, 24105 Kiel, Germany

^2^Department of Pathology, University of Hannover, 30625 Hannover, Germany

^3^Department of Biological Sciences and Department of Chemistry, Columbia University of New York, NY 10027, USA

^4^The Walter and Eliza Hall Institute of Medical Research, Parkville, Victoria 3052, Australia

^5^Department of Medical Biology, University of Melbourne, Parkville, Victoria 3052, Australia

**Keywords** Ferroptosis, ACSL4, necroptosis, MLKL, ischemia-reperfusion injury

***Corresponding author:**

Stefan Krautwald (Ph.D.)

University Hospital Schleswig-Holstein, Campus Kiel

Department of Nephrology and Hypertension

Georges-Köhler-Haus

Fleckenstr. 4

24105 Kiel

Germany

Phone: +49-431-500-23040

Fax: +49-431-500-23044

E-mail: krautwald@nephro.uni-kiel.de

web: http://www.nephrologie-uni-kiel.de

**Supplementary Figure S1**

*Acsl4*- and *Mlkl*-knockout in different human cell lines protects from ferroptosis and necroptosis, respectively. **a** Five different *Acsl4*-silenced clones of the human fibrosarcoma cell line HT-1080, referred to as HT-1080-A1 to HT-1080-A5 and **(b)** likewise five different *Mlkl*-silenced clones of the human colon adenocarcinoma cell line HT-29, referred to as HT-29-M1 to HT-29-M5 were analyzed by Western blotting at protein level for the ablation of the indicated target genes. Lysates of the mock-transfected (non-edited) control cells were loading in each case in the left lane. Both blots were re-developed with an antibody against β-actin as a loading control. All positive evaluated clones were tested successful once for insensitivity of erastin-induced ferroptotic cell death (HT-1080-A1, HT-1080-A2, and HT-1080-A3) and TSZ-induced necroptotic cell death (HT-29-M1, HT-29-M2, HT-29-M4, and HT-29-M5), respectively, to confirm the success of the knockout also at cellular level. **(c-e)** NIH3T3 cells are not only susceptible to erastin- but also to RSL3-induced ferroptosis. FACS analysis for the necrotic marker 7-AAD and phosphatidylserine exposure (annexin V-FITC positivity) in **(c)** parental (not-edited) NIH3T3 cells, **(d)** *Acsl4*-edited NIH3T3 clone NIH-A1, and **(e)** *Mlkl*-edited NIH3T3 clone NIH-M1 which were treated for 16 hrs at 37°C with DMSO (vehicle), 2 µM RSL3, and 2 µM RSL3 + 1 µM ferrostatin-1 (RSL3 + Fer-1) as indicated. Depicted is one of four independent experiments. **f** Results obtained by flow cytometry enumeration of necroptotic and ferroptotic cell death in the genetically unmodified parental NIH3T3, the *Acsl4*-edited NIH3T3 clone NIH-A1 and the *Mlkl*-edited NIH3T3 clone NIH-M1, respectively, that have been already illustrated in Figure 1c-e were verified by measuring loss of plasma membrane integrity using LDH release assays. Mean ± SD is shown for four independent experiments. Notably, LDH release from a single experiment was calculated from the average of three identically treated wells. **g** To rule out the possibility that loss of ACSL4 expression might be a universal protective mechanism used by cancer cells to avoid their own demise by ferroptosis we checked the general expression of ACSL4 in genetically unmodified ferroptotic-insensitive HT-29 and L929 cells by Western blotting. All represented cell lines and *Acsl4*-edited NIH3T3 clones that serve as internal negative control were left untreated and equal amounts of protein (20 µg/lane) were resolved by SDS/PAGE. The expression of ACSL4 (M_r_ = 79.0 kDa) was performed with a specific rabbit monoclonal ACSL4 antibody (abcam) and visualized by using enhanced chemiluminescence (ECL). The blot was stripped and re-probed with an antibody against β-actin (Cell Signaling) as loading control (at the bottom).

**Supplementary Figure S2**

Increased sensitivity to erastin-mediated ferroptosis is specific for MLKL inhibition. **a** FACS analysis of parental NIH3T3 cells that were pretreated as indicated for 30 min with DMSO (vehicle), 50 µM Nec-1_s_, 1 µM GSK’872 or 50 µM dabrafenib, respectively. Ferroptosis was induced thereafter for 12 hrs by the addition of 10 µM erastin (era). Notably, unlike with pretreatment of the parental NIH3T3 cells with the murine MLKL inhibitor GW806742X, we did not observe increased sensitivity to erastin-mediated ferroptosis after pretreatment with the stable variant of the RIPK1 inhibitor necrostatin-1 (Nec-1_s_) or the RIPK3-kinase inhibitors, GSK’872 and dabrafenib. Depicted is one of three independent experiments. **b** The overlay images show detection of accumulated ROS over time in parental (red) vs. *Mlkl*-edited NIH3T3 cells (blue), respectively. The cells were treated for indicated time at 37°C with 10 µM erastin. Notably, earlier stimulation points as depicted in **(a)** are required to clearly illustrate ROS production as central signal event of ferroptosis. Depicted is one of three independent experiments.

**Supplementary Figure S3**

Ferroptosis and necroptosis are intertwined at the protein level in a functionally relevant *in vivo* model of murine IRI. **(a-d)** Male C57BL/6 wildtype mice and *Mlkl*-knockout mice underwent 35 min of bilateral pedicle clamping (ischemia) followed by different times of reperfusion. Serum creatinine and urea concentrations as well as histological consideration of organ damage were determined as illustrated in Fig. 4, respectively, after the mice were sacrificed at the indicated reperfusion time points. **a** Different expression levels of ACSL4 (79.0 kDa) and **(b)** MLKL (54.0 kDa) were distinguishable in whole-kidney lysates taken from wildtype or *Mlkl*-knockout mice during the time course of IRI. **c** Stripping of the blot and reprobing with a murine phospho-specific MLKL antibody (abcam) in this head-to-head study indicated a lack of reliable detection of phosphorylated MLKL (pMLKL) in this tissue. **d** The blot was stripped again and re-probed with an antibody against β-actin (Cell Signaling) as loading control (at the bottom). **e** The suitability and specificity of the murine anti-phospho-MLKL antibody is demonstrated with cultured cell samples *in vitro*. Parental NIH3T3 cells were treated for 6 hrs at 37°C with DMSO (vehicle), 100 ng/ml TNFα + 25 µM zVAD (TZ), and 100 ng/ml TNFα + 25 µM zVAD + 2.5 µM GW806742X, as indicated. Cells were pretreated as indicated for 30 min at 37°C with GW806742X to block MLKL-dependent necroptosis. Equal amounts of protein (20 µg/lane) were resolved by reducing SDS/PAGE and detection of phosphorylated MLKL (M_r_ = 54.0 kDa) was done with the identical antibody that was used before in **(c)**.

**Supplementary Figure S4**

Histological changes confirm that ferroptosis and necroptosis are intertwined in murine IRI. **(a-c)** Male C57BL/6 wildtype mice (wt) and *Mlkl*-knockout mice (*Mlkl*-ko) underwent 35 min of bilateral pedicle clamping (ischemia) followed by different times of reperfusion. Representative histological sections of murine kidney biopsies obtained from untreated groups as well as 6, 12, and 24 hrs after onset of reperfusion were stained with specific antibodies (see above) as indicated for the presence of **(a)** ACSL4, **(b)** MLKL and **(c)** phospho-MLKL after sacrificing the mice at the indicated reperfusion time points (n = 4 animals for each group and time point, scale bars = 50 µm).
